# Bridging between NMA and Elastic Network Models: Preserving All-Atom Accuracy in Coarse-Grained Models

**DOI:** 10.1371/journal.pcbi.1004542

**Published:** 2015-10-16

**Authors:** Hyuntae Na, Robert L. Jernigan, Guang Song

**Affiliations:** 1 Department of Computer Science, Iowa State University, Ames, Iowa, United States of America; 2 Department of Biochemistry, Biophysics and Molecular Biology, Iowa State University, Ames, Iowa, United States of America; 3 Program of Bioinformatics and Computational Biology, Iowa State University, Ames, Iowa, United States of America; 4 L. H. Baker Center for Bioinformatics and Biological Statistics, Iowa State University, Ames, Iowa, United States of America; UNC Charlotte, UNITED STATES

## Abstract

Dynamics can provide deep insights into the functional mechanisms of proteins and protein complexes. For large protein complexes such as GroEL/GroES with more than 8,000 residues, obtaining a fine-grained all-atom description of its normal mode motions can be computationally prohibitive and is often unnecessary. For this reason, coarse-grained models have been used successfully. However, most existing coarse-grained models use extremely simple potentials to represent the interactions within the coarse-grained structures and as a result, the dynamics obtained for the coarse-grained structures may not always be fully realistic. There is a gap between the quality of the dynamics of the coarse-grained structures given by all-atom models and that by coarse-grained models. In this work, we resolve an important question in protein dynamics computations—how can we efficiently construct coarse-grained models whose description of the dynamics of the coarse-grained structures remains as accurate as that given by all-atom models? Our method takes advantage of the sparseness of the Hessian matrix and achieves a high efficiency with a novel iterative matrix projection approach. The result is highly significant since it can provide descriptions of normal mode motions at an all-atom level of accuracy even for the largest biomolecular complexes. The application of our method to GroEL/GroES offers new insights into the mechanism of this biologically important chaperonin, such as that the conformational transitions of this protein complex in its functional cycle are even more strongly connected to the first few lowest frequency modes than with other coarse-grained models.

## Author Summary

Proteins and other biomolecules are not static but are constantly in motion. Moreover, they possess intrinsic collective motion patterns that are tightly linked to their functions. Thus, an accurate and detailed description of their motions can provide deep insights into their functional mechanisms. For large protein complexes with hundreds of thousands of atoms or more, an atomic level description of the motions can be computationally prohibitive, and so coarse-grained models with fewer structural details are often used instead. However, there can be a big gap between the quality of motions derived from atomic models and those from coarse-grained models. In this work, we solve an important problem in protein dynamics studies: how to preserve the atomic-level accuracy in describing molecular motions while using coarse-grained models? We accomplish this by developing a novel iterative matrix projection method that dramatically speeds up the computations. This method is significant since it promises accurate descriptions of protein motions approaching an all-atom level even for the largest biomolecular complexes. Results shown here for a large molecular chaperonin demonstrate how this can provide new insights into its functional process.

## Introduction

Protein dynamics plays a key role in describing the function of most proteins and protein complexes. The importance of protein dynamics studies has been increasingly recognized alongside the importance of the structures themselves. Experimentally, protein dynamics can be studied using nuclear magnetic resonance (NMR) [1, 2], time-resolved crystallography [3], fluorescence resonance energy transfer (FRET) [4] and other single-molecule techniques [5], etc. Computationally, the study of protein dynamics most commonly relies upon molecular dynamics (MD) simulations [6–8]. Normal mode analysis (NMA) is another popular and powerful tool for studying protein dynamics and was first applied to proteins in the early 80’s [9–11]. The advantage of normal modes over MD is that they can most efficiently describe protein motions near the native state. To apply NMA, a structure is first energetically minimized. The minimized structure is then used to construct the Hessian matrix, from which normal modes can be obtained from its eigenvectors and eigen-frequencies. This method poses a huge demand on computational resources, especially memory, since some large supramolecules may have hundreds of thousands of atoms. The time spent on computing the eigenvalues/eigenvectors also is large, of the order of the cube of the number of atoms. Consequently, its applications are limited to smaller systems.

For this reason, many simplified models [12–33] have been developed for efficient normal mode computations. These models use simplified structural models or simplified force fields or commonly, both. One commonly applied type of coarse-grained model is the elastic network model [13, 16], which usually treats each residue as one node, and residue-residue interactions as Hookean springs. It has been demonstrated for a large number of cases that these extremely simplified models can still capture quite well the slow dynamics of a protein [12]. And because of their high level of simplicity, they have been successfully applied to study the normal mode motions of the largest structural complexes such as GroEL/GroES [18, 34–38], ribosome [22, 39–41], nuclear pore complex [29], etc.

However, along with the significant gains from this simplicity comes also some loss of accuracy, particularly in the accuracy of the normal modes [42, 43]. The validity of most simplified models was justified *a posteriori*, by comparing with experimental B-factors or sets of multiple experimental structures for example. How well they preserve the accuracy of the original NMA has rarely been assessed directly [33]. To overcome this problem of accuracy, we built a strong connection between NMA and elastic network models (ENMs) through a series of steps of simplification that began with NMA and ended with ENMs, and proposed a new way to derive accurate elastic network models in a top-down manner (by gradually simplifying NMA) [33]. Our derivation was based on the realization that the Hessian matrix of the original NMA can be written as a summation of two main terms, the spring-based terms and the force/torque-based terms, with the former contributing significantly more than the latter. By ignoring the latter term, we obtained at a new model, sbNMA (or spring-based NMA), that has high accuracy and closely resembles the original NMA and requires no energy minimization. sbNMA, like the original NMA, is force-field dependent and uses many parameters. By further simplifying it, we arrived at two force-field independent elastic network models, ssNMA (simplified spring-based NMA) and eANM (enhanced ANM), both of which use many fewer parameters and yet still preserve most of the accuracy of NMA [33]. For example, the mean square fluctuations predicted by ssNMA for a set of small to medium proteins have an average correlation of nearly 0.9 with those predicted with the original NMA [33]. It was shown [42] also that ssNMA modes are more accurate than those from other elastic network models. However, this bridging, as detailed in Ref. 33, connected NMA only with all-atom elastic network models but not with coarse-grained ones. Both ssNMA and eANM, though strongly resembling NMA, are by nature all-atom models and cannot be directly applied to coarse-grained structures.

There is little doubt that for very large biomolecular systems, coarse-grained structure representations are needed, since all-atom normal mode analyses for such systems are computationally often out of reach. Our aim in this work is to extend the idea of bridging between NMA and elastic network models to coarse-grained models while preserving sufficient accuracy to obtain accurate protein dynamics even for very large systems. Is it possible to efficiently construct coarse-grained models whose description of the dynamics of a coarse-grained structure remains as accurate as that given by all-atom models? Coarse-grained models, such as C^*α*^-based models, obviously do not have all the structural details of all-atom models. But can they produce the dynamics of the C^*α*^ atoms as accurately as all-atom models? Is it possible to have *both* the simplicity of coarse-grained structures and the accuracy of all-atom interactions? These questions are the focus of this work. And we demonstrate affirmative answers to these questions by employing a novel iterative matrix projection technique.

While our earlier work [33] connects between NMA and all-atom elastic network models and represents a force-field simplification of NMA while maintaining most of its accuracy, the present work presents an additional structural simplification from all-atom elastic network models to coarse-grained elastic network models. Combined together, the two pieces of work provide a bridge between all-atom NMA and coarse-grained elastic network models and should reveal deep insights for how to develop coarse-grained elastic network models that preserve most of the accuracy of all-atom NMA.

## Methods

A coarse-grained model has two key components: i) a coarse-grained structure representation, and ii) an interaction model for the coarse-grained structure. The challenge that one normally faces in developing coarse-grained models is that there is no prescription for how to represent the interactions among the coarse-grained structure *precisely* [44]. Most semi-empirical force field potentials are for atomic models. Highly simplified Hookean springs were commonly used to model residue-residue interactions. They provide only a very rough approximation to the atomic interactions. Other studies that link atomic and coarse-grained models apply force-matching [44] or require their frequency spectra to have similar distributions [45]. A statistical mechanical foundation was developed by the same research group [46] to show that many-body potentials of mean force that govern the motions of the coarse-grained sites could be generated. Regarding coarse-grained structure representation, C^*α*^ atoms are normally used to represent residues, although other coarse-grained representations also have been investigated [47].

In this work, to extend the accurate all-atom models to coarse-grained models without losing accuracy in the dynamics, we take two steps. First, we show that it is possible to define a precise interaction model for the coarse-grained structure so that its dynamics are the same as that of its all-atom counterpart. Second, we show that the construction of such a precise interaction model can be performed efficiently and straightforwardly.

### How to Construct a Precise Interaction Model for a Coarse-Grained Structure?

It is useful first to perform an operation that separates out the atoms used for the coarse-graining from the remainder of the atoms. Mathematically, it is possible to define a *precise* interaction model (in the form of a Hessian matrix) for the coarse-grained structure by first rearranging the original Hessian matrix **H**_*all*_ into parts for the coarse-grained atoms and the remainder of the atoms in separate subspaces, as was done by Eom et. al. [48] and Zhou and Siegelbaum [49]:

$$H_{all}=(\begin{array}{cc} H_{cc} & H_{cr}\\ H_{cr}^{⊤} & H_{rr} \end{array}),$$ (1)

$${\tilde{H}}_{cc}=H_{cc}-H_{cr}H_{rr}^{-1}H_{rc}^{⊤},$$ (2)

where *c* stands for the atoms used for the coarse-graining, *r* stands for the residual part of the structure, and ⊤ represents the matrix transpose. It can be shown mathematically [50, 51] that ${\tilde{H}}_{cc}$ maintains the same description of the mean-square fluctuations and cross-correlations of the coarse-grained structure as the original Hessian matrix. All elements in ${\tilde{H}}_{cc}^{-1}$ are the same as their corresponding elements in $H_{all}^{-1}$. A similar idea of using matrix projection to obtain the motions for subsystems was previously used also by Brooks and Zheng and their co-workers [52, 53] to develop their VSA (vibration subsystem analysis) model.

However, this mathematical rearrangement in Eq (2) requires the inversion of **H**_*rr*_, which appears to be nearly as difficult as computing the inverse of the original all-atom Hessian matrix, assuming the number of atoms in the coarse-grained structure is much smaller than that of the original all-atom model. Therefore, unless ${\tilde{H}}_{cc}$ can be computed in an efficient way, the precise interaction model defined in Eq (2) would be computationally too expensive to apply for very large systems and thus of little practical utility.

In the next section, we present a novel way for computing ${\tilde{H}}_{cc}$ efficiently, without directly inverting **H**_*all*_ or **H**_*rr*_. As a result, this permits an efficient construction of coarse-grained models that can represent the dynamics of the coarse-grained structure as accurately as all-atom models.

### Efficiently Construct the Coarse-Grained Hessian Matrix through Iterative Projection

To efficiently obtain the Hessian matrix ${\tilde{H}}_{cc}$ from Eq (2) without having to directly invert **H**_*rr*_, we take advantage of the fact that the Hessian matrix **H**_*all*_, the second derivatives of the potential, can be highly sparse for some all-atom models. **H**_*all*_ is not so sparse for the conventional NMA, due to the persistence of electrostatic interactions to long distances. However, it is sparse for ssNMA, an accurate all-atom model that closely resembles NMA as mentioned above.

The potential for ssNMA includes most of the same interaction terms as for NMA, except for the electrostatic interactions [33]. As a simplified model of spring-based NMA (or sbNMA), ssNMA uses one single uniform spring constant for all bond stretching terms, one uniform spring constant for all the bond-bending terms, and one for the torsional terms. Its non-bonded van der Waals interactions are truncated near the equilibrium distance to avoid negative spring constants in the Hessian matrix [33]. A single set of van der Waals radii are used for all van der Waals interactions. All the equilibrium values such as bond lengths, bond angles, and torsional angles are taken from the reference structure. Consequently, most of the off-diagonal elements in the ssNMA Hessian matrix are zero.

In the following, we use ssNMA to construct the all-atom Hessian matrix **H**_*all*_ and show how a precise interaction model ${\tilde{H}}_{cc}$ can be efficiently constructed through an iterative matrix projection procedure. We call this model coarse-grained ssNMA, or CG-ssNMA. CG-ssNMA preserves the same accuracy as the all-atom ssNMA in its description of the dynamics of the coarse-grained structure.

The procedure, as detailed below, takes full advantage of the sparseness of the Hessian matrix. Given a protein that has *n* atoms, one can iteratively reduce its size (or coarse-grain it) by removing one atom, or a group of *r* atoms, at a time without losing accuracy in depicting the motions of the remaining atoms. This can be done by adding a correction term to the interactions among the remaining atoms. Define by **H** the Hessian matrix with *n* atoms as follows:

$$H=(\begin{array}{cc} H_{kk} & H_{kr}\\ H_{kr}^{⊤} & H_{rr} \end{array}),$$ (3)

where **H**_*kk*_ is the block matrix of **H** for the kept *n* − *r* atoms, **H**_*rr*_ the block matrix for *r* atoms to be removed, and **H**_*kr*_ represents the interactions between the group of atoms to be removed and the remaining atoms. The effective Hessian matrix ${\tilde{H}}_{kk}$ of the kept atoms after taking into account the correction term can be written as [42, 48, 49]:

$${\tilde{H}}_{kk}=H_{kk}-H_{kr}H_{rr}^{-1}H_{kr}^{⊤},$$ (4)

with $H_{kr}H_{rr}^{-1}H_{kr}^{⊤}$ being the correction term.

It can be shown that the motions of the remaining atoms as described by ${\tilde{H}}_{kk}$ is the same as from the original Hessian matrix **H**. This numerical preservation is crucial when an all-atom Hessian matrix is gradually coarse-grained by repeatedly removing non-C^*α*^ atoms, since it guarantees that the quality of the description of the C^*α*^ atoms remains the same while the size of the Hessian matrix is being reduced.

Note that each atom interacts only with a few, say *m* on average, atoms due the sparseness of the Hessian matrix. As a result, **H**_*kr*_ has only a small number (*rm*) of non-zero elements, representing the interactions between the group of atoms to be removed and the kept atoms. Therefore, the term $H_{kr}H_{rr}^{-1}H_{kr}^{⊤}$ in Eq (4) can be computed in *O*(*r* ^3^ + *r* ^2^ *m* ^2^) time. Coarse-graining the whole protein structure takes roughly *n*/*r* iterations and thus requires a total time of *O*((*r* ^2^ + *rm* ^2^)*n*), which is *linear* in the protein size *n*.

To further reduce the running time, matrix elements that are near zero (weak interactions) are set to zero if their absolute values are less than a predetermined threshold value *ξ*. A properly chosen *ξ* can further improve computation speed while preserving the accuracy, by effectively reducing the number of interactions, especially those between the atoms being removed and the remaining atoms. Different *ξ* values are tested, as detailed in the next section.

**Fig 1:**
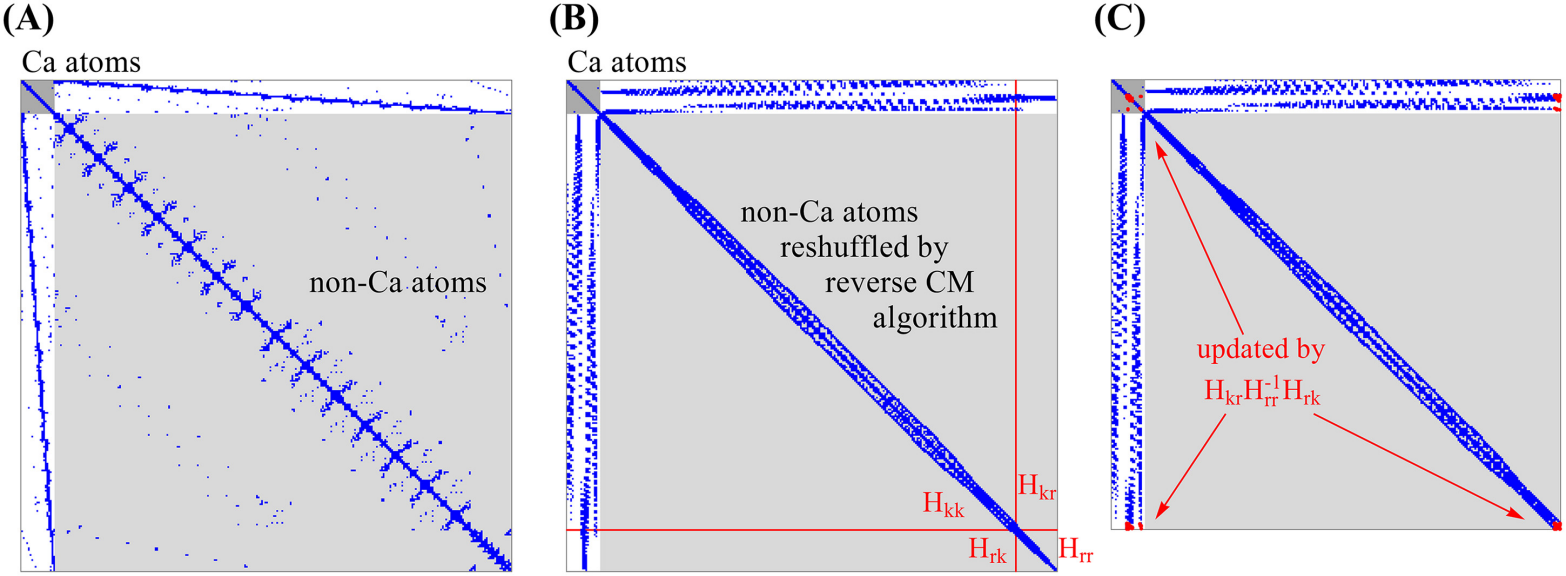
Illustration of how the sparseness of the Hessian matrix can be maintained throughout the iterative matrix projection procedure, when coarse-graining is performed by selecting the C^*α*^ atoms for retention. (A) In the first step the original Hessian matrix is shuffled so that C^*α*^ atoms (in dark gray at the top-left corner) are separated from the non-C^*α*^ atoms (in light gray). Blue dots represent non-zero elements. (B) In the second step the non-C^*α*^ atoms are rearranged again so that those interacting with one another are placed close together in the matrix using, for example, the Cuthill-McKee algorithm [54]. As a result, most non-zero elements are placed near the diagonal. (C) Matrix after performing one projection to remove atoms in group *r*. The red dots represent the blocks modified by the projection. The sparseness of the non-C^*α*^ region is mostly unaffected. The sparseness of the white region (interactions with C^*α*^ atoms) can be maintained by using an appropriate threshold value ξ, see text.

Fig 1 illustrates how the sparseness of the Hessian matrix is maintained throughout the iterative matrix projection procedure. At the initial step, atoms are shuffled so that C^*α*^ atoms are grouped together and placed on the left-most side of the Hessian matrix, as shown in Fig 1(A), where the grouped C^*α*^ and non-C^*α*^ atoms are represented by dark and light gray blocks, respectively. Blue dots represent the non-zero elements of the Hessian matrix. The non-C^*α*^ atoms can then be further rearranged, for example, using the Cuthill-McKee algorithm [54], so that the atoms that interact with one another are placed close together in the matrix. As a result, the non-zero elements are relocated near the diagonal of the matrix (see Fig 1(B)). In such a sparse matrix, Fig 1(C) shows the effect of applying one matrix projection using Eq (4), where the red dots represent the elements of the matrix whose values are modified. Note that the sparseness of the non-C^*α*^ region is mostly unaffected by the projection. The sparseness of the white region (interactions with C^*α*^ atoms) can be maintained by using an appropriate threshold value *ξ* mentioned earlier.

Algorithm 1 lists the steps that iteratively reduce the all-atom Hessian matrix to a coarse-grained one. The algorithm takes as input the all-atom Hessian matrix **H**, a set of C^*α*^ atom indices {*k*_1_, …, *k*_*n*_}, and a threshold value *ξ*. All matrix elements whose absolute values are less than *ξ* are set to 0. In practice, it turns out that lines 4–11 run more efficiently if each iteration of the coarse-graining process removes not a single atom but a group of atoms (*R*_*i*_ as in line 2). Removing a group of adjacent atoms reduces the average number of interactions (*m* in the above Big-O notation) with the remaining atoms. These groups of atoms are determined by spatially partitioning the whole structure (3-D) into cubic blocks (18~Å for each dimension). These blocks represent initial groups of atoms. The reason why atoms are partitioned in this way is to minimize the number of interactions among the different groups. Blocks are then sorted by their sizes (i.e., the number of atoms) in descending order. Next, starting with the smallest one, blocks on the “small” end (usually blocks on the outsides of a structure) are iteratively merged together with the next smallest block as long as the size of the merged group does not exceed the size limit (which is about 500 atoms per group, the number of atoms in a regular cubic block). The merging process stops when there are no small blocks left to be merged. In lines 7 and 9, sparse(**A**, *b*) returns a sparse matrix of **A** by setting to zero **A**’s elements that satisfy |**A**_*i,j*_| < *b*, where |**A**_*i,j*_| is the absolute value of **A**_*i,j*_. Threshold *ξ*/*m* is used in line 9 since the addition (or subtraction) in line 10 is accumulated *m* times. Line 9 prevents very small values from being added to **H** in line 10 and then removed in line 7 at the next iteration.

**Algorithm 1** CoarseGrain(**H**, {*k*_1_, …, *k*_*n*_}, *ξ*)

1: *K* ← {*k*_1_, …, *k*_*n*_}

2: *R* ← {*R*_1_, *R*_2_, …, *R*_*m*_}

3: **H** ← Hessian matrix of **H** reshaped in the order of *K*, *R*_1_, *R*_2_, …, *R_m_*

4: **for** *i* = *m*, *m* − 1, …, 1 **do**

5:  $k\leftarrow \left|K\right|+\sum_{j=1}^{i-1}\left|R_{j}\right|$

6:  *r* ← *k* + ∣*R*_*i*_∣

7:  **B** ← sparse(**H**_1..*k, k* + 1..*r*_, *ξ*)

8:  **D** ← **H**_*k* + 1..*r*, *k* + 1..*r*_

9:  **E** ← sparse(**BD** ^−1^ **B** ^⊤^, *ξ*/*m*)

10:  **H**_1..*k*,1..*k*_ ← **H**_1..*k*, 1..*k*_ − **E**

11: **end for**

12: **H** ← sparse(**H**_1..|*K*|,1..|*K*|_, *ξ*)

13: **return H**

## Results

### Validation of Model Accuracy and Efficiency

In this section, we first verify computationally that the coarse-grained ssNMA model constructed according to the proposed procedure indeed not only preserves the accuracy of all-atom models in its description of the motions of the coarse-grained structure but also is computationally efficient. To this end, we first show, by applying it to a dataset of 177 small to medium proteins, that with a properly chosen threshold value *ξ*, the coarse-grained ssNMA preserves full accuracy. We then extend the same coarse-graining procedure, using the same *ξ* value, to construct coarse-grained ssNMA Hessian matrices for 80 large superamolecules of different sizes and show that the construction of these ssNMA Hessian matrices requires only a nearly linear time and can thus be carried out quickly, even for large systems.

### The Iterative Coarse-Graining Procedure Preserves Accuracy

To validate the accuracy of the method, Algorithm 1 is applied to 177 small-to-medium sized proteins whose sizes are greater or equal to 60 residues but less than 150. This is the same set of proteins that was used in our earlier work [33]. Only small to medium sized proteins are used at this stage due to the high computational costs of running all-atom models, which have also been computed here for comparison purposes.

Each protein structure is first energy minimized. From the all-atom ssNMA Hessian matrix, two coarse-grained Hessian matrices, **H** and $\hat{H}$, are computed. **H** is computed by direct matrix projection (as in Eq (2)), which is an exact but very expensive computation, while $\hat{H}$ is computed with the proposed iterative projections as in Algorithm 1. To show that $\hat{H}$ preserves the same accuracy as **H**, we compute the correlations between mean square fluctuations (MSF) computed with **H** and those with $\hat{H}$, and the eigenvalue-weighted overlaps between modes by **H** and those by $\hat{H}$. The eigenvalue-weighted mode overlap is defined as:

$$\sum_{i=7}^{3n}\frac{w_{i}}{w}\left|m_{i}\cdot {\hat{m}}_{i}\right|,$$ (5)

where *n* is the number of atoms, **m**_*i*_ (and ${\hat{m}}_{i}$) is the *i*th mode of **H** (and $\hat{H}$), *w*_*i*_ = 1/*λ*_*i*_ is the relative weight and is set to be the inverse of the *i*th eigenvalue of **H**, and $w=\sum_{i=7}^{3n}w_{i}$ is the normalization factor. The reason why we use the modes with the same indices (**m**_*i*_ and ${\hat{m}}_{i}$) instead of the best matching modes when computing the weighted-overlap is to measure also how well the order of the modes is preserved. Lower frequency modes are given higher weights in this weighted overlap measure. The intuition behind this weighted mode scheme is that it represents how similar the modes (including their orders) are between the two models.

Table 1 shows the levels of accuracy that can be achieved when different threshold values *ξ* are applied to ssNMA [33]. It is seen that ssNMA preserves the full accuracy (1.0 in correlations and overlaps) in mean square fluctuations and modes when a threshold value (*ξ*) as large as 0.01 is used. Similar results are also seen for the enhanced ANM model (eANM) [33], another all-atom model that closely resembles NMA. Using a large threshold value allows the sparseness of the Hessian matrix to be maintained during the iterative matrix projection process and consequently the construction of the coarse-grained ssNMA Hessian matrix to be carried out quickly.

**Table 1:** The accuracy of models at different threshold values *ξ*.

*ξ* ^a^	NMA (0.0^b^)		ssNMA (0.99^b^)		eANM (0.98^b^)	
	corr^c^	w-ovlp^d^	corr	w-ovlp	corr	w-ovlp
0.0001	0.99	0.96	1.00	1.00	1.00	1.00
0.001	0.85	0.62	1.00	1.00	1.00	1.00
0.01	0.82	0.69	1.00	1.00	1.00	0.99
0.1	0.56	0.53	0.99	0.92	0.98	0.83

The accuracy of ssNMA, in both mean-square fluctuations and mode details, is fully preserved at *ξ* = 0.01. The initial sparseness of the Hessian matrix, in parentheses, is 0.0, 0.99, 0.98 for NMA, ssNMA, and eANM, respectively.

^a^ *ξ*: the threshold value used to set to zero the small elements in the Hessian matrix;

^b^initial sparseness of the Hessian matrix;

^c^corr: mean-square fluctuation correlation;

^d^w-ovlp: eigenvalue-weighted mode overlap as defined in Eq (5).

For conventional NMA, however, the iterative coarse-Graining approach as described above does not work nearly as well (see Table 1). This is due to the slowly-decreasing, long-range electrostatic interactions.

### The Iterative Coarse-graining Procedure Is Efficient

Secondly, we look at the efficiency, i.e., how much time does this iterative coarse-graining procedure require? To this end, we apply the same iterative coarse-graining procedure to construct coarse-grained ssNMA Hessian matrices for a number of large proteins and protein complexes. The same threshold value, *ξ* = 0.01, is used, which has been shown in the previous section to preserve the full accuracy.

**Fig 2:**
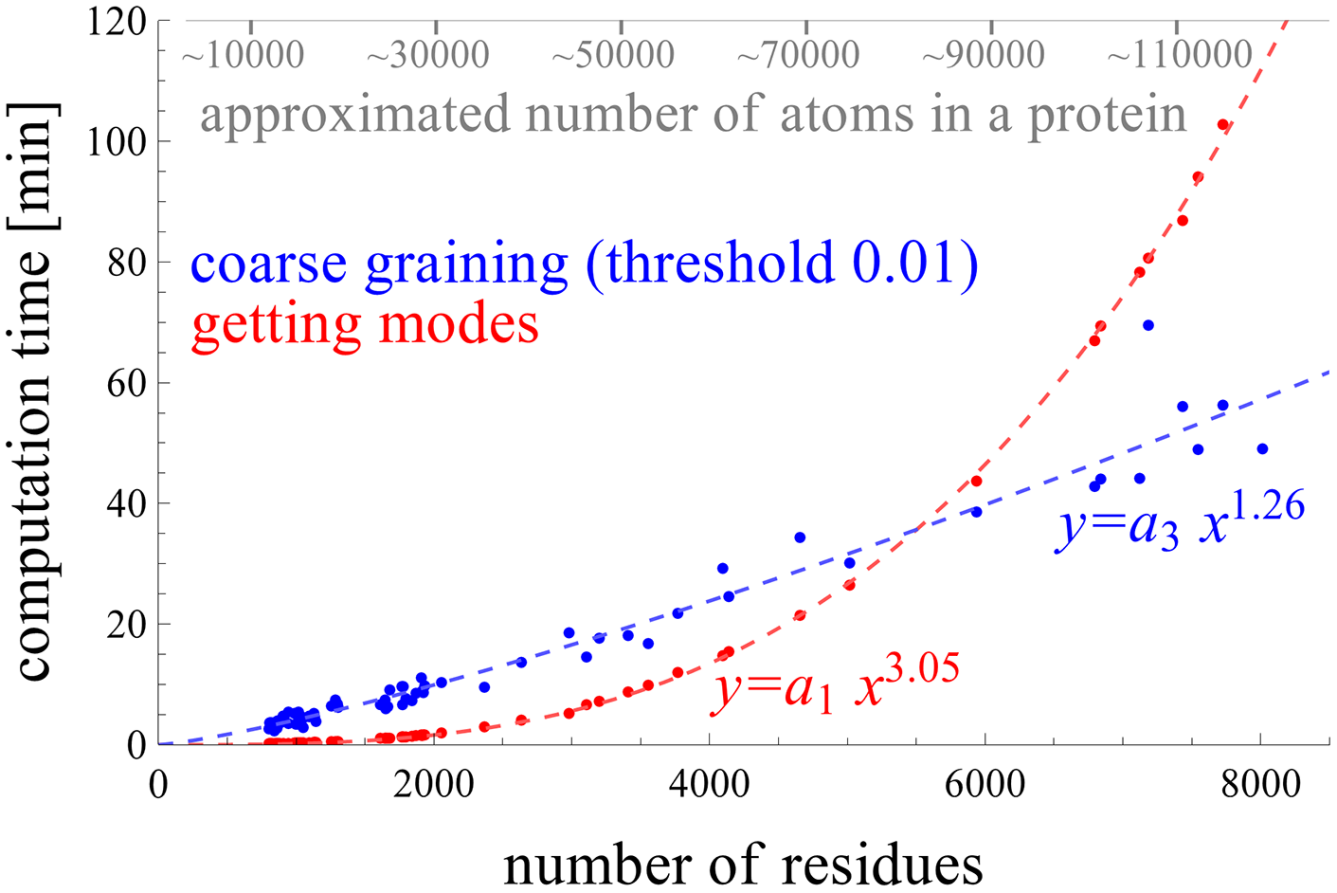
Comparison of the coarse-graining time using the proposed method and the diagonalization time of the coarse-grained Hessian matrix.

Fig 2 shows the efficiency (computational time) of the proposed method as a function of the system size. In the figure, each blue and red point represent respectively, for a protein of that size, the coarse-graining time, i.e., the time required to construct the coarse-grained ssNMA Hessian matrix (with *ξ* = 0.01), and the diagonalization time of that coarse-grained Hessian matrix. The dashed lines show the growth rates of the time cost as a function of the system size. The curves are obtained from the least squares fitting to a non-linear function *f*(*x*) = *ax* ^*b*^. As shown in the figure, the diagonalization time (red curve) grows approximately as the cube, while the coarse-graining time grows approximately linearly. Especially for large complexes, the time needed for coarse-graining the all-atom Hessian matrix using Algorithm 1 becomes increasingly smaller relative to the diagonalization time. As a result, the total time for computing the normal modes for such large protein complexes using the coarse-grained ssNMA Hessian matrices is about the same as for other coarse-grained elastic network models such as ANM.

In summary, the results in this section demonstrate that the proposed iterative coarse-graining procedure not only preserves the accuracy in depicting the motions of the coarse-grained structures but is also computationally highly efficient.

This result is significant since it means that we can construct coarse-grained models that preserve all-atom accuracy even for very large protein complexes, which was not previously possible. Next, as an application, we apply the proposed procedure to compute and analyze the dynamics of the GroEL/GroES complex.

### Application to GroEL/GroES complex

**Fig 3:**
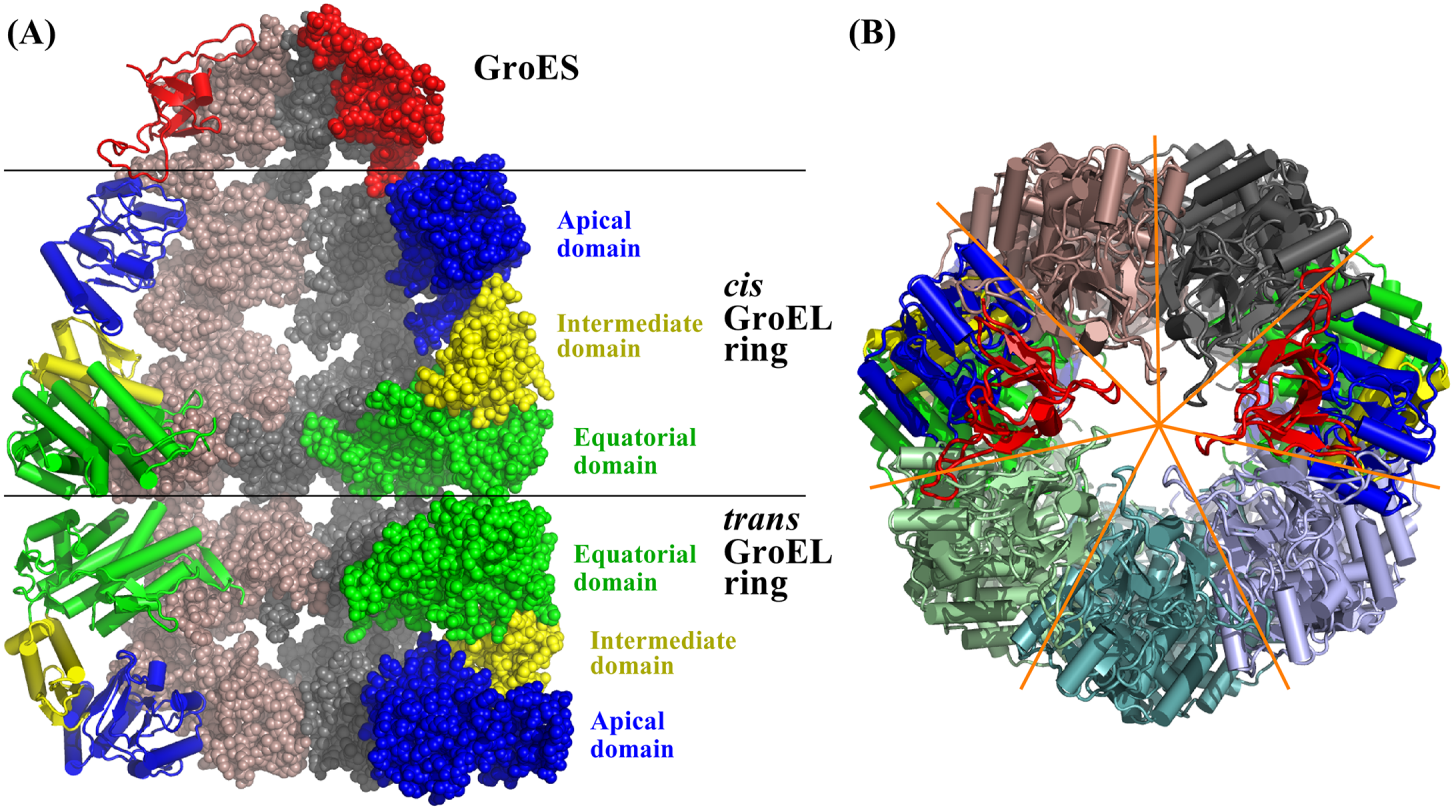
Structure of the GroEL/GroES complex in (A) front and (B) top views. For subunits of the GroEL, the equatorial, intermediate, and apical domains of cis and *trans* rings are colored green, yellow, and blue, respectively. The GroES cap is displayed in red.

The GroEL/GroES complex [55] is a molecular chaperone that assists the unfolding of partially folded or misfolded proteins, by providing them with the chance to refold. GroEL consists of *cis* and *trans* rings, each of which has 7 subunits. Each subunit is 547 residues. GroES also has 7 chains and each chain contains 97 residues. The GroEL *cis*-ring and GroES form a capped chamber that can hold proteins and facilitate protein unfolding partly through their intrinsic collective motions, such as compressing, stretching, twisting, shearing, and relaxing. Fig 3 shows the GroEL/GroES structure (pdbid: 1AON) in top and front views. In Fig 3(A), the three domains of the *cis* and *trans* rings are distinguished with different colors: equatorial (green), intermediate (yellow), and apical (blue) domains.

To understand its functional mechanisms, it is informative to obtain the intrinsic motions of this complex. However, for large protein complexes such as GroEL/GroES that has over 8,000 residues, standard all-atom NMA will take a prohibitively large memory and a long time to run. Consequently, past normal mode studies on this complex were limited to coarse-grained models [18, 36], or all-atom models of single subunits [34]. Though a more accurate description of its normal modes is highly desirable and may provide deeper insights into the functional mechanism of the complex, it was lacking due to computational constraints.

Here, we apply the proposed iterative procedure to obtaine a coarse-grained ssNMA Hessian matrix for the entire GroEL/GroES complex. This coarse-grained ssNMA (or CG-ssNMA) model preserves the same all-atom accuracy in its description of the motions of the coarse-grained structure as the original ssNMA.

### Mean-Square Fluctuations

First, we apply CG-ssNMA to compute mean-square fluctuations. To this end, we use the GroEL-GroES-(ADP)_7_ complex (pdbid: 1AON) [55] as the initial structure. This structure is composed of the co-chaperone GroES, the *cis*-ring whose subunits are bound with 7 ADPs, and the *trans*-ring (see Fig 3).

#### Structure Preparation

The residues whose side-chains are not present in the PDB structure (1AON) are effectively treated as alanines (no side chains have been added). Since the crystal structure contains only heavy atoms, hydrogen atoms are added using the psfgen program from VMD [56] and energetically minimized. Lastly, the Hessian matrix of all-atom ssNMA [33] is determined, and is coarse-grained using the proposed procedure as detailed in Algorithm 1.

**Fig 4:**
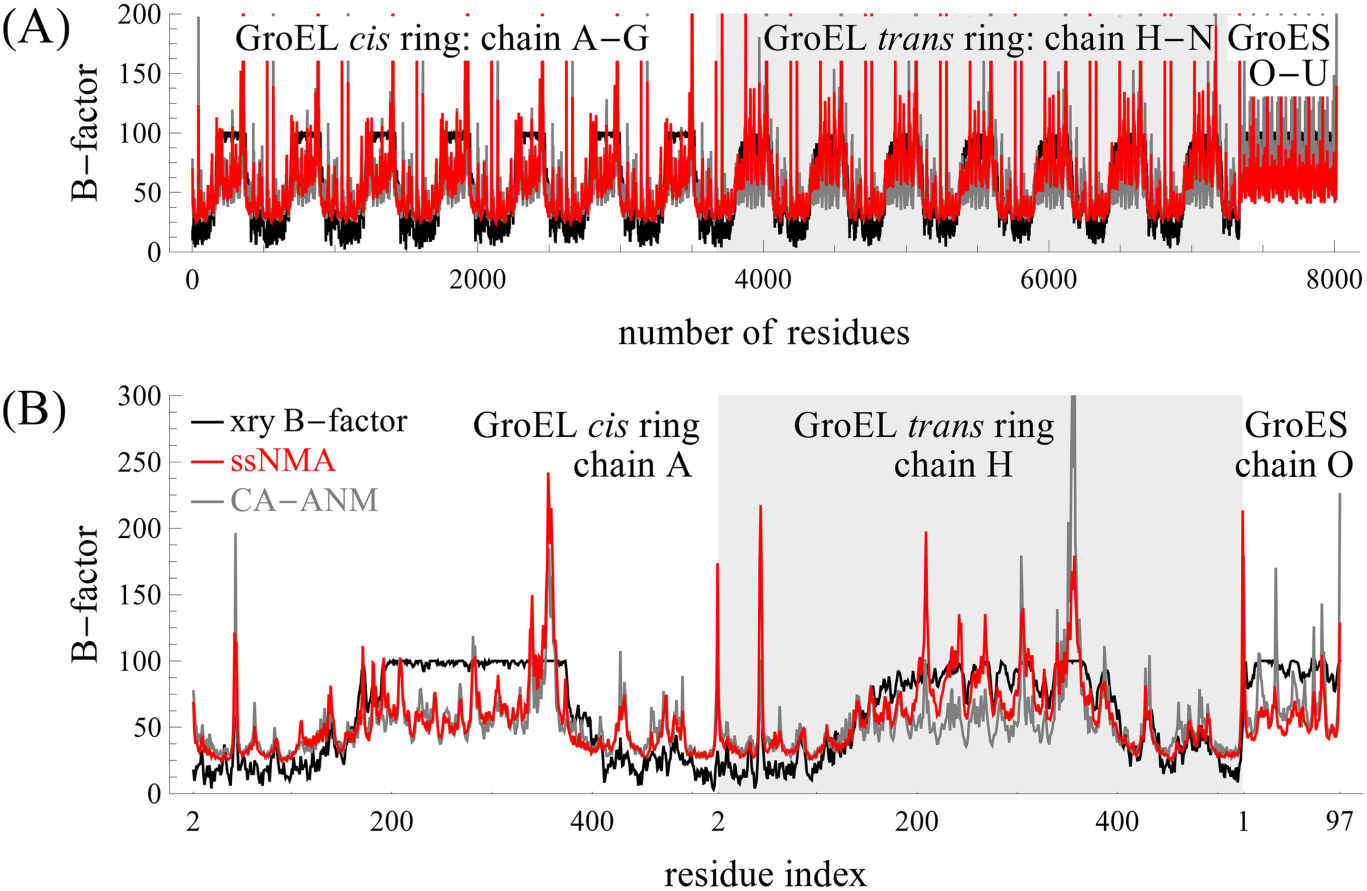
Comparisons of the experimental B-factors with the mean-square fluctuations (MSFs) computed with the new coarse-grained ssNMA and with ANM, for (A) all residues and (B) only the first subunit in each ring. The middle gray region is the *trans*-ring of GroEL, and the left and right white regions are the *cis*-ring of GroEL and the GroES cap, respectively.

Fig 4 shows the mean-square fluctuations (MSFs) determined by CG-ssNMA (in red) and by the coarse-grained C^*α*^-based ANM (in gray), and the experimental B-factors (in black). In (A), all 8015 residues’ MSFs and B-factors are shown for three separate parts: the *cis*-ring with a white background, the *trans*-ring with a light gray background, and the GroES cap with a white background. In (B), the first subunits of the three parts (*cis* and *trans* rings, and GroES) are re-plotted to show the MSF in more detail. In the figure, the mean-square fluctuations by ssNMA and ANM are computed using all the modes (including all the high-frequency modes) and scaled to minimize the root-mean-square deviation from the experimental B-factors. The correlation between experimental and predicted B-factors is 0.69 for ssNMA, and 0.52 for ANM. Note that there are a few high peaks in ssNMA MSFs.

### Motion Correlations and Cooperativity

**Fig 5:**
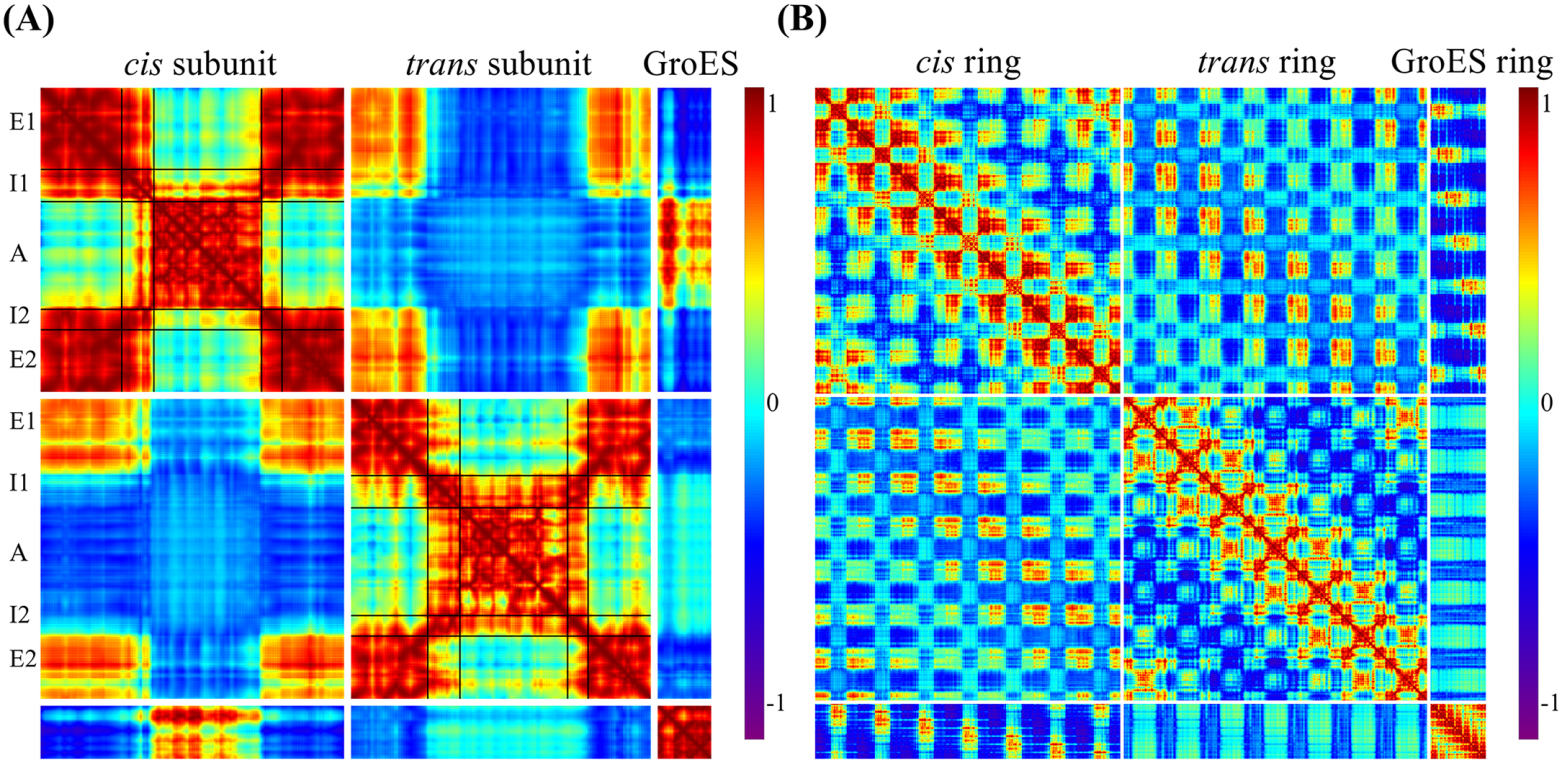
Cooperativity of residue motions using the first 15 lowest frequency modes of the coarse-grained ssNMA model. (A) The cooperativity within a single set of subunits: chain A from the cis ring, chain H from the *trans* ring, and chain O from GroES. (B) The cooperativity among all residue pairs in the GroEL/GroES complex.

The motion correlation (or cooperativity) *C*_*i*,*j*_ between the *i*-th and *j*-th residues can be expressed as follows:

$$C_{i,j}=\frac{\left\langle r_{i}\cdot r_{j}\right\rangle }{{\left(\left\langle r_{i}\cdot r_{i}\right\rangle \left\langle r_{j}\cdot r_{j}\right\rangle \right)}^{1/2}},$$ (6)

where **r**_*i*_ and **r**_*j*_ are the displacement vectors for the *i*-th and *j*-th residues in a given mode, respectively, **a** ⋅ **b** is the dot product of two vectors **a** and **b**, and ⟨*a*⟩ is the average value of *a* within the first *k* lowest frequency modes. Fig 5 shows the cooperativity of residue motions within each subunit and across the whole protein complex. The cooperativity plot is generated from the first 15 dominant (i.e., lowest frequency) modes given by the coarse-grained ssNMA.

Fig 5(A) shows the cooperativity among residue pairs within a single set of subunits: one subunit from the *cis* ring (chain A of 1AON), one from the *trans* ring (chain N), and one from GroES (chain O). The cooperativity of residue pairs is color coded: red for strong correlated motions (*C*_*i*,*j*_ = 1), cyan for uncorrelated (*C*_*i*,*j*_ = 0), and purple/blue for anti-correlated (*C*_*i*,*j*_ = −1). The most noticeable difference between the *cis* and *trans* rings is the involvement of the intermediate domain in the motions of the apical or equatorial domain. In the *cis* ring, the red regions indicate that the motions of intermediate domain (I1 and I2) are strongly correlated with those of the equatorial domain (E1 and E2), while the motions of the apical domain (A) are largely independent of them. In the *trans* ring, however, the motions of intermediate domains (I1’ and I2’) are more correlated with those of the apical domain (A’) than with the equatorial domain (E1’ and E2’). A similar cooperativity plot for the ANM model is given in Supporting information (S1 Fig). Overall, the two methods give similar correlation patterns. The main noticeable difference is that the relative motions between equatorial (E1’ and E2’) and apical (A’) domains of the trans-ring subunit are more clearly shown as anti-correlated (i.e., the region appears to be bluer) in Fig 5 (given by ssNMA) than with ANM shown in S1 Fig.

One general role of the intermediate domain is connecting the apical and equatorial domains and facilitating the communication between them. The results in Fig 5 imply that the dynamics or motion partner of the intermediate domain depends on the structural form of the GroEL ring: *cis* or *trans*. Considering the structure transitions of *cis* → *trans* and *trans* → *cis* that take place during the GroEL/GroES functional cycle, it is not surprising that the transition path in the former case may be different from a simple reverse of the latter. Additionally, Fig 5(A) shows that the motions of GroES and the apical domain (A) of the *cis* ring also are highly correlated.

The cooperativity of all the residues in the complex is presented in Fig 5(B). Along the off-diagonal there are four dark blue mesh bands, implying that the apical domains of the subunits that sit on opposite sides across the rings, such as chain C/D and chain A, are strongly anti-correlated. Another interesting observation is that the motions of GroES are strongly anti-correlated to the equatorial domain of the *cis* ring.

### The Characteristics and Quality of the ssNMA Modes

The ssNMA model presented in this work, though coarse-grained in structure, maintains an all-atom level accuracy in its description of the interactions and consequently an all-atom level accuracy in its description of the normal mode motions of the coarse-grained structure. Such an accurate description of the normal mode motions is highly desirable but has not been performed before for large protein complexes such as GroEL/GroES that has over 8,000 residues. In the following, we will examine closely the first few lowest frequency modes of ssNMA and characterize their motions. The quality of these modes is then assessed. A comparison with C^*α*^-based ANM modes is made at the end.

**Fig 6:**
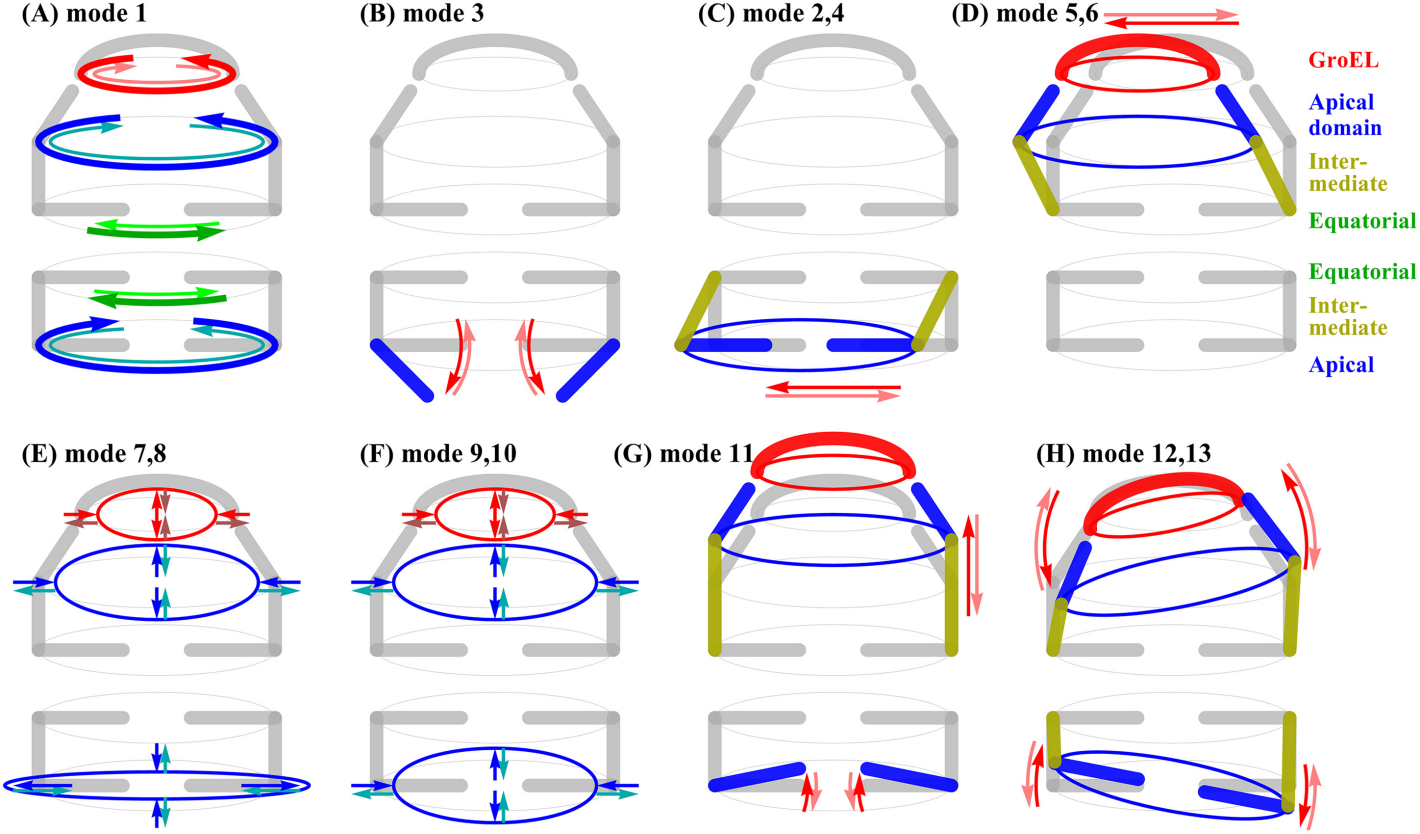
Descriptions of the first 13 lowest frequency modes of GroEL/GroES, determined by the coarse-grained ssNMA. (A) mode 1, (B) mode 3, (C) modes 2 and 4, (D) modes 5 and 6, (E) modes 7 and 8, (F) modes 9 and 10, (G) modes 11, and (H) modes 12 and 13.

Fig 6 characterizes the slow dynamics of GroEL/GroES in individual modes or pairs of modes. The first lowest frequency mode portrays a rotational motion around the cylindrical axis of the complex. This mode matches with the first mode of ANM nearly perfectly, with a high overlap of 0.97. The third mode is about opening the gate of the *trans* ring to receive substrates into its chamber, by moving its apical domains to conform its structure to resemble that of the *cis* ring. The second and fourth modes are mainly about a swing motion of the *trans* ring. This motion also helps to open the chamber gate of the *trans* ring. In ssNMA, this gate opening motion in the *trans* ring is clearly captured by these three distinct modes, especially the third mode, whose importance is manifested also in the conformation transitions during the GroEL/GroES functional cycle that will be described in the next section. In ANM, there is not a single mode that closely matches the third mode of ssNMA. The gating opening motion seems to spread into several modes in ANM and be mingled with other motions. The 5th–6th modes are shearing motions of the GroES cap and the apical domains of the *cis* ring. This motion causes them to shift significantly relative to the equatorial domains. This motion (in the 5th/6th modes) is similar, to some extent, to that in the second and third modes of ANM, which in turn have some resemblance also to the second/fourth modes of ssNMA. The 7th–10th modes display alternating motions of compression and extension of the whole complex. The 11th mode is mainly about stretching/compressing the chamber of the *cis*-ring. To some extent, this motion (of the 11th mode) changes the structure of the *cis* ring towards the shape of the *trans* ring. The 12th–13th modes are mainly about tilting the *cis*/*trans* rings and the GroES cap.

The animations of the top 13 dominant modes (lowest frequency) of ssNMA (and ANM) are made available at http://www.cs.iastate.edu/~gsong/CSB/coarse.

Next, we compare more quantitatively the modes of ssNMA and ANM.

#### Quantitative comparisons of the normal modes of ssNMA and ANM

Table 2 summarizes the overlaps between the lowest frequency modes of coarse-grained ssNMA and ANM. Note that the first ssNMA mode matches nearly perfectly with the 1st ANM mode with a high overlap value of 0.97, while other modes match only moderately well. The order of modes between the two models also seems to be scrambled. The fairly low overlap values indicate that only the lowest frequency mode is well preserved in ANM, but significantly less so for other modes. This is consistent with our previous observations [42, 57]. The third ssNMA mode is mainly about opening the gate of the *trans* ring by moving its apical domains apart so that its structure becomes more similar to the *cis* ring. This mode is functionally important as it describes a key protein transition (see the next section). However, in ANM, the closest resemblance of this motion is to the 20th mode that describes a mixed motion of expanding/compressing of both GroEL chambers.

**Table 2:** ssNMA modes and their corresponding best matching modes in ANM with which they have the largest overlaps.

ssNMA	ANM	overlap	ssNMA	ANM	overlap
1	1	0.97	8	7	0.83
2	4	0.65	9	9	0.64
3	20	0.62	10	10	0.66
4	5	0.66	11	8	0.78
5	2	0.68	12	11	0.60
6	3	0.72	13	12	0.62
7	6	0.77			

Results shown are for the first 13 lowest frequency modes, the same modes whose motion characteristics are presented in Fig 6.

**Fig 7:**
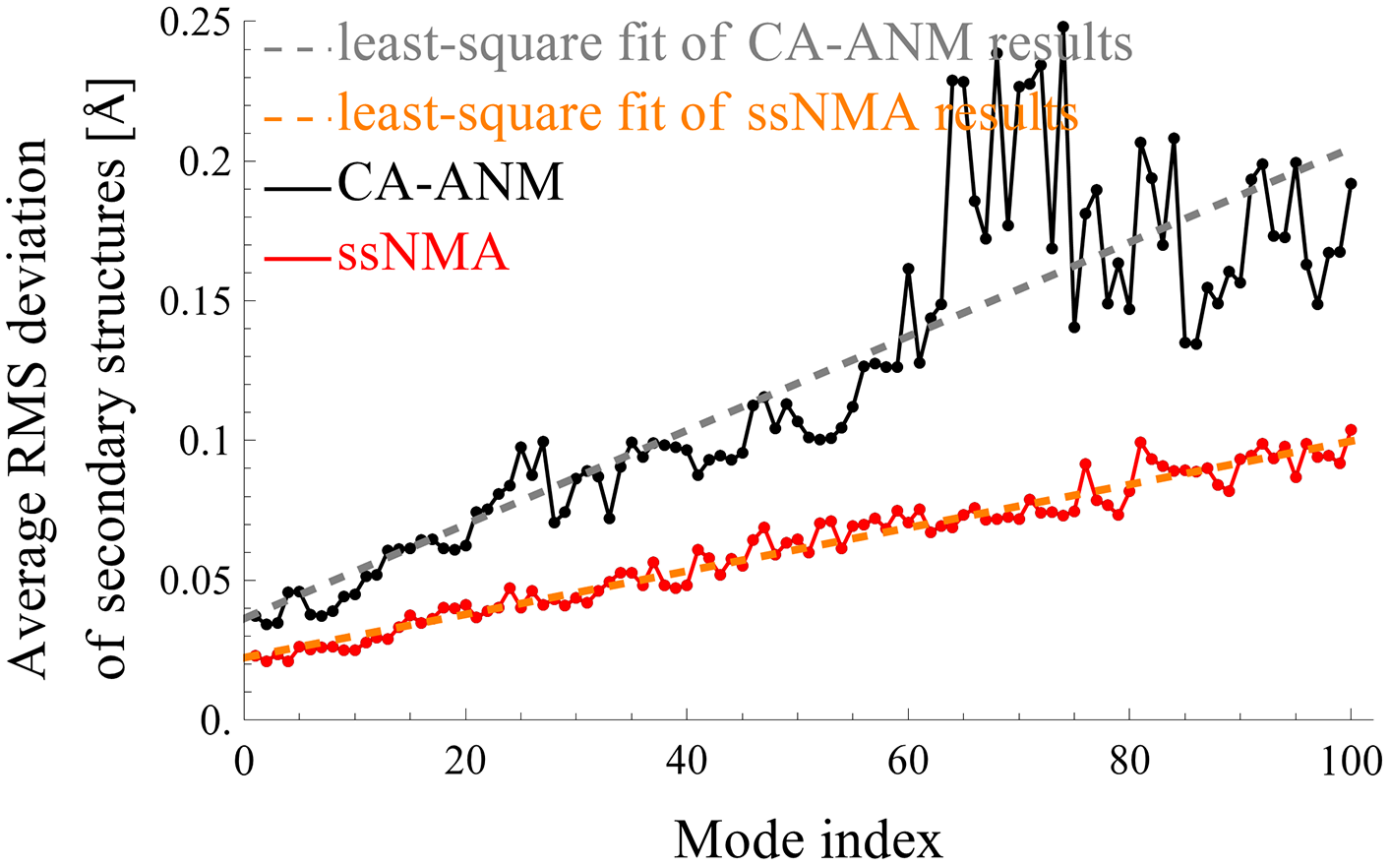
Preservation of secondary structures in mode motions. The solid red (or black) line represents the average structure deviations of all secondary structures of the GroEL/GroES complex when it moves along a normal mode of ssNMA (or ANM). The dashed lines are the least-square fits to the solid lines.

Fig 7 shows how well the quality of the secondary structures are preserved as the protein complex moves in the directions of the modes of ssNMA or ANM. In this study, for each mode, the protein structure is deformed along the mode direction until its RMSD changes 1 Å from the initial structure. The RMSDs of individual secondary structures (alpha-helices or beta-sheets) are determined independently, and the average RMSDs of these secondary structures are then computed. This procedure is repeated for the first 100 lowest frequency modes of both coarse-grained ssNMA and ANM. In the figure, the solid red (black) line represents the secondary structure deviations by the coarse-grained ssNMA (or ANM), and the dashed lines are the least-square fits to the solid lines. The plot shows that secondary structures are preserved about twice as well with ssNMA as with ANM.

In summary, there are two major quality improvements in ssNMA modes over ANM modes, both of which can be attributed to the all-atom accuracy that is maintained in ssNMA. First, the secondary structures are better preserved in ssNMA modes than in ANM. The modes determined by coarse-grained ssNMA appear to be more accurate and realistic. This is consistent with the the more realistic potential that ssNMA employs. ssNMA has several terms in its potential function that enforce covalent geometry while the ANM model treats the whole system with uniform elastic springs. Second, which is related to the first, the modes by coarse-grained ssNMA seem to characterize the different collective motion patterns of the protein complex better. So, interestingly there is some significant amount of cohesion that is lost in the coarse-graining with ANM but is retained in CG-ssNMA.

### Normal Models Facilitate the Functional Conformation Transitions

In this section, we apply CG-ssNMA to interpret the conformation transitions in the functional cycle of GroEL/GroES. Our hypothesis is that the intrinsic normal mode motions of the complex should facilitate its conformation transitions. To measure how well the modes are related to the conformation transitions, we compute the overlaps between normal modes and a given transition. We then repeat the computations and analysis using ANM and compare the results with those from CG-ssNMA.

In total there are six conformation transitions among the five known conformation states of the complex (see Table 3) considered: T → R, T → R′′′, $R^{\prime \prime }\to R_{\text{flipped}}^{\prime \prime }$, $R_{\text{nocap}}^{\prime \prime }\to R_{\text{nocap,flipped}}^{\prime \prime }$, R′′ → S, and S → R′′, where “nocap” stands for the absence of the GroES cap. Table 4 summarizes, for these transitions, the top 3 largest overlaps found using CG-ssNMA and ANM. The indices of the modes that give the largest overlaps also are given. The first two cases represent transitions from the apo form to ATP/GroES bound forms. The transitions $R^{\prime \prime }\to R_{\text{flipped}}^{\prime \prime }$ and $R_{\text{nocap}}^{\prime \prime }\to R_{\text{nocap,flipped}}^{\prime \prime }$ were thought to take place during the functional cycle of GroEL/GroES [58], in which the two GroEL rings alternate as a functional chaperone. However, recent work [59] suggested that in *vivo* the GroEL/GroES complex assumes a football shape in the functional process and that both GroELs might work simultaneously as protein unfolding chaperones. For this reason, we consider also the functional transitions between states R′′ and S. Table 4 lists the results.

**Table 3:** The five conformation states of the GroEL/GroES complex used in this work.

conformation	pdb-id	description
T state	1GR5	The tense state.
R	2C7E	The relaxed state, 7 ATP bound
R′′	1AON	Bullet-shaped structure, 7 ADP bound, GroES bound
${\text{R}}_{*}^{\prime \prime }$	1GRU	Bound with 7 ATP and 7 ADP, GroES bound
S	4PKO	S is obtained by removing one GroES ring from the football-shaped complex 4PKO that is bound with two GroES rings and 14 ADP.

**Table 4:** Top three highest overlaps between structure displacements and normal modes computed by CG-ssNMA or C^*α*^-based ANM.

transition models	T → R		$\text{T}\to {\text{R}}_{*}^{\prime \prime }$		${\text{R}}^{\prime \prime }\to {\text{R}}_{\text{flipped}}^{\prime \prime }$		${\text{R}}_{\text{nocap}}^{\prime \prime }\to {\text{R}}_{\text{nocap,flipped}}^{\prime \prime }$		R′′ → S		S → R′′	
	ovlp	mode	ovlp	mode	ovlp	mode	ovlp	mode	ovlp	mode	ovlp	mode
coarse-grained ssNMA	0.56	4	0.64	4	0.40	11	0.49	17	0.53	1	0.41	2
	0.24	46	0.27	60	0.36	15	0.29	4	0.39	3	0.26	3
	0.24	16	0.25	17	0.26	3	0.29	13	0.27	15	0.24	14
CA-ANM	0.57	1	0.55	1	0.46	13	0.51	18	0.52	1	0.47	3
	0.33	51	0.40	11	0.36	32	0.43	17	0.35	20	0.39	14
	0.33	10	0.23	96	0.33	20	0.27	116	0.26	68	0.24	24

Structure S is obtained by removing one GroES from the football-shaped structure (pdbid: 4PKO). For the transition from R′′ to its flipped counterpart, the normal modes are computed either with or without the GroES cap, in both of these cases only the two GroEL rings are used to compute the conformation displacement. The values in each table entry are the overlaps between the given conformation transition and a mode, with the mode index also given.

**T → R and** $T\to R_{*}^{\prime \prime }$: Transitions T → R and $R_{*}^{\prime \prime }$ in Table 4 show that these are mostly achieved with a torsional motion along the vertical axis of the structure. Both the CG-ssNMA and ANM models capture this torsional motion, but their mode indices are different. It is the fourth mode in CG-ssNMA that gives the largest overlap while it is the first in ANM. The results clearly show that the motion to R (as induced by ATP binding) is along the path to $R_{*}^{\prime \prime }$, as observed by Roseman et al. [60] from low resolution cryo-EM images.

**$R^{\prime \prime }\to R_{\text{flipped}}^{\prime \prime }$:** Ranson et al. [58] suggested that the functional process of GroEL/GroES involves alternations to the two GroEL rings as functional units and the complex is bullet-shaped [55] in *vivo*.

Here we consider the transition from a bullet-shaped complex (R′′) to its flipped counterpart. In this transition, one of the GroEL rings goes from the *trans* form to the *cis* form, while the other ring changes from *cis* to *trans*. Results in Table 4 show that the coarse-grained ssNMA captures well the transition from *trans* to *cis* using its fourth mode, which has the second largest overlap, while the 17th mode has the best overlap and characterizes mostly the transition from *cis* to *trans* ring, as well as a partial transition from *trans* to *cis*. ANM, on the other hand, describes the transition of *trans* → *cis* and *cis* → *trans* using the 17th and 18th modes, each of which is a mixture of both *cis*-ring and *trans*-ring deformations.

It is thought that after the binding of the ATPs to the trans ring, the GroES cap is removed and the substrate protein is released. Then the two GroEL rings go through *trans* → *cis* and *cis* → *trans* transitions, respectively, and another GroES will bind the opposite ring, completing a cycle. The GroES cap stabilizes the *cis* ring in its conformation and prevents its transition to a *trans* conformation. However, after the ATP binding at the opposite ring, the GroES cap is removed, which makes the transition from a *cis* to a *trans* conformation easier. The larger overlap seen in this transition without the GroES cap (see Table 4) provides evidence that GroES is probably removed first before the *cis* ↔ *trans* conformation transitions take place rather than occurring simultaneously. This agrees with the idea that structures facilitate functional transitions.

**R′′ → S (opening the *trans* ring gate):** Recent work by Fei et al. [59] suggested that the GroEL/GroES complex in *vivo* should have a football shape. The formation of a football-shaped GroEL/GroES complex was thought to be promoted by substrate protein (SP), and that “SP shifts the equilibrium between the footballs and bullets in favor of the former, consequently making them the predominant species.” [59]

Here, we examine the transitions between a football-shaped complex and a bullet-shaped complex. Transition R′′ → S opens the gate of the *trans* ring to receive a substrate protein (unfolded or misfolded) in its chamber. This is accomplished by conforming the structure of its apical domain to that of a *cis* ring (see the third mode in Fig 6 and in S1 Video). S2 Fig highlights the conformation change that takes place within a *trans*-ring monomer in this transition. The overlaps between the transition and normal modes reveal a large contribution by the torsional rotation along the vertical axis (mode 1), as the *trans* ring of S is rotated about 8 degree counter-clockwise from that of R′′ [59]. Secondly, this transition is captured by the third ssNMA mode that mainly depicts a chamber-opening motion. In contrast, CA-ANM provides this transition mainly using its 20th mode, which is a mixture of the chamber opening motion and some other deformation of the *cis* ring and the GroES cap.

**S → R′′ (closing the *cis* ring gate):** Transition S → R′′ closes the gate of the *cis* ring to conform its structure to that of a *trans* ring. Similar to the transition R′′ → S, this transition requires torsional rotations and gate-closing motions. The coarse-grained ssNMA captures this transition using the second and third low frequency modes. CA-ANM captures the torsional rotation properly using the third mode, but has to rely on higher-frequency modes to capture the gate-closing transition (See Table 4, last column).

#### Summary

For all the above conformation transitions, CG-ssNMA’s interpretation of them involves more of the first few lowest frequency modes than ANM. This is consistent with the observation made earlier that ssNMA modes tend to preserve the secondary structures better and thus likely are of better quality. Indeed, it is expected that the all-atom accuracy that CG-ssNMA maintains should render a more accurate description of protein motions.

## Discussion

Normal mode analysis (NMA) is an indispensable tool for obtaining the patterns of intrinsic collective dynamics of biomolecular systems around their native states. Such dynamics studies and computations are important since dynamics is tightly linked to functional mechanisms and can reveal insights that studies based on static structures alone cannot provide. For very large complexes and eventually even a cell, all-atom descriptions of the dynamics of the system are neither feasible nor necessary. A coarse-grained structure representation is often sufficient. But what about the dynamics for a coarse-grained structure? Even though the structure representation is coarse-grained, we still would like to have an accurate description of its dynamics, ideally as close in accuracy to an all-atom model as possible.

It was by the use of coarse-grained models that past normal mode studies of very large biomolecular systems were carried out and remarkable insights were gained in these studies [18, 22, 29, 36, 39, 40]. There is little doubt that the levels of coarse-graining chosen for studying these large systems were appropriate. However, what was not previously assessed was the quality of the dynamics that was provided by those coarse-grained models. Since most coarse-grained models use extremely simple potentials to model the interactions within the coarse-grained structures, the dynamics they render are likely to have some deficiencies.

In this work, we have successfully bridged this gap and have presented a new method for constructing coarse-grained models that can preserve all-atom accuracy in dynamics. The method takes advantage of the sparseness of the Hessian matrix and iteratively reduces its size through projection until it is reduced to that of the desired coarse-grained structure. Since the projections maintain the accuracy of the interactions, the final Hessian matrix represents the precise interactions within the coarse-grained structure. Compared with the RTB (rotation-translation block) method [61] or BNM (block normal modes) [19], which assumes rigidity and ignores flexibility within each block, our method provides a more accurate description of the motions of the coarse-grained systems. Compared with the VSA model (vibration subsystem analysis) [52, 53], the advantage of our method is that it is computationally significantly more efficient.

Results presented in this work are highly significant since they promise to provide descriptions of normal mode motions at the all-atom level of accuracy even for the largest biomolecule complexes. While preserving all-atom accuracy through matrix projection is not new and has been done previously [49, 50, 52, 53], one of our key contributions here is developing a new algorithm that can carry out this matrix projection highly efficiently and therefore make it applicable to very large structure complexes, which has not been done previously. Such accurate descriptions of the intrinsic dynamics may help reveal new insights into the functional mechanisms of many biomolecular systems. It should be noted that because we are able to efficiently obtain a precise interaction model (the Hessian matrix) for the coarse-grained systems, we can solve it not only for the first few lowest frequency modes, but for all the modes. This is in line with the overarching aim of our work: to bridge between NMA and coarse-grained elastic network models while preserving the all-atom accuracy. If only the first few lowest frequency modes are needed, then there are some alternative methods that may be more efficient.

Our application of the method to GroEL/GroES reveals some new insights into the functional process of this biologically important chaperonin. For example, our results show that the conformational transitions of this protein complex in its functional cycle are even more closely linked to the low frequency modes than was previously observed using other coarse-grained models.

This work is a continuation of our previous work that aimed to bridge NMA with elastic network models [33]. While the previous work bridged between NMA and all-atom elastic network models, this work represents the second half of developing this bridge, namely between *all-atom* elastic network models and *coarse-grained* elastic network models. Combined together, the two pieces of work demonstrate how one can bridge between the conventional NMA that uses an all-atom model with a full force-field and coarse-grained elastic network models that are nowadays the preferred choice for normal mode computations due to their simplicity. This bridging reveals novel insights on how one may develop coarse-grained models that are not only simple to use, but also maintain most of the accuracy of the conventional NMA.

### Limitations and Future Work

Although the proposed iterative coarse-graining procedure can be used to efficiently construct coarse-grained models whose description of the dynamics of the coarse-grained structures preserves all-atom accuracy, it is limited in that it can be applied only to some of the models, such as ssNMA, eANM, or sbNMA (see S1 Table). It cannot be applied to the conventional NMA. This is because the potential of NMA contains electrostatic interactions that decay rather slowly and consequently the NMA Hessian matrix is not sparse; however, there remain some uncertainties about how to best compute the electrostatics.

A possible partial solution is to add a switch function to the non-bonded interactions of NMA and make it decay to zero at some cutoff distance, as is commonly done in MD simulations. This will make the Hessian matrix much sparser and make it possible to apply the proposed iterative procedure to NMA. We have shown this to be the case (see results in S1 Table). However, this is only a partial solution since it recovers only the short range part of the electrostatics. The long range electrostatic interactions, which may have a pronounced contribution to long-range collective motions and cooperativity, are still missing. Additionally, the cumbersome energy minimization (which ssNMA does not require) becomes necessary, which can be a challenge when working with large biomolecular complexes.

One possible future work is to study the effects of electrostatic interactions on normal modes, specifically the extent of contributions by short-range or long-range electrostatic interactions. If the short-range component of the electrostatic interactions dominates the long range component in contributing to normal modes, then the aforementioned partial solution will provide an excellent approximation.

## Supporting Information

S1 Table The accuracy of screened-NMA and sbNMA at different threshold values *ξ*. (DOCX)

S1 Fig Cooperativity of residue motions using the first 15 lowest frequency modes of the CA-ANM model. (A) The cooperativity within a single set of subunits: chain A from the *cis* ring, chain H from the *trans* ring, and chain O from GroES. (B) The cooperativity among all residue pairs in the GroEL/GroES complex.
(EPS)

S2 Fig The conformation changes within a trans-ring subunit in R′′ → S transition. The *trans*-ring subunit of conformation R′′ is represented by the thin gray line, while that of conformation S by the thin red line. The thick curve (in blue, yellow, and green) displays, for this R′′ → S transition, the conformation change contributed by the third mode (of the ssNMA model) alone. This figure shows that a large conformation change takes place within the subunits in this conformation transition and is well captured by the third mode of ssNMA. The three conformations shown are aligned at the equatorial domain (in green).
(EPS)

S1 Video The important gate-opening mode (mode 3) in R′′ → S transition. The video shows the motions of the GroEL/GroES complex along this important gate-opening mode. More animations for transitions listed in Table 4 are available at http://www.cs.iastate.edu/~gsong/CSB/coarse.
(WMV)
