# Supplementary material for: Bridging between NMA and Elastic Network Models: Preserving All-Atom Accuracy in Coarse-Grained Models
Source: PLoS Comput Biol. 2015 Oct 16;11(10):e1004542. doi: 10.1371/journal.pcbi.1004542 (PMC4608564; doi:10.1371/journal.pcbi.1004542)
Supplement: S1 Table — (DOCX) [file pcbi.1004542.s001.docx]

S1 Table. The accuracy of screened-NMA and sbNMA at different threshold values $\xi$.

| $\xi^{a}$ | screened-NMA^b^ (0.87^c^) | | sbNMA (0.99^c^) | |
| --- | --- | --- | --- | --- |
|  | corr^d^ | w-ovlp^e^ | corr | w-ovlp |
| 0.0001 | 1.00 | 1.00 | 1.00 | 1.00 |
| 0.001 | 1.00 | 1.00 | 1.00 | 1.00 |
| 0.01 | 1.00 | 0.98 | 1.00 | 1.00 |
| 0.1 | 0.95 | 0.62 | 0.99 | 0.92 |

This table is an extension of Table 1 and contains results for two more models: screened-NMA and sbNMA, whose accuracy also is (nearly) fully preserved at $\xi=0.01$. The initial sparseness of the Hessian matrix, in parentheses, is 0.87 and 0.99 for screened-NMA and sbNMA, respectively.

(All the following remarks except b are the same as those in Table 1.)

1. the threshold value used to set to zero the small elements in a Hessian matrix;
2. screened-NMA: same as NMA except that its non-bonded interactions (electrostatics and van der Waals) are tapered to zero at 9.0 Å.
3. initial sparseness of the Hessian matrix;
4. corr: mean-square fluctuation correlation;
5. w-ovlp: eigenvalue-weighted best-mode overlap as defined in Eq. (5):

$$\sum_{i=7}^{3n} \frac{w_{i}}{w}\left| \mathbf{m}_{i}\cdot{\hat{\mathbf{m}}}_{i} \right|,$$

where $n$ is the number of atoms, $\mathbf{m}_{i}$ (and ${\hat{\mathbf{m}}}_{i}$) is the $i$th mode of $\mathbf{H}$ (and $\hat{\mathbf{H}}$), $w_{i}=1/{\lambda_{i}}$ is the relative weight and is set to be the inverse of the $i$th eigenvalue of $\mathbf{H}$, and $w=\sum_{i=7}^{3n} w_{i}$ is the normalization factor;
